# Sustained Photosynthetic Performance of *Coffea* spp. under Long-Term Enhanced [CO_2_]

**DOI:** 10.1371/journal.pone.0082712

**Published:** 2013-12-06

**Authors:** José C. Ramalho, Ana P. Rodrigues, José N. Semedo, Isabel P. Pais, Lima D. Martins, Maria C. Simões-Costa, António E. Leitão, Ana S. Fortunato, Paula Batista-Santos, Isabel M. Palos, Marcelo A. Tomaz, Paula Scotti-Campos, Fernando C. Lidon, Fábio M. DaMatta

**Affiliations:** 1 Grupo Interações Planta-Ambiente - Plant Stress, Centro de Ambiente, Agricultura e Desenvolvimento - BioTrop, Instituto de Investigação Científica Tropical, I.P., Oeiras, Portugal; 2 Centro de Estudos Florestais, Instituto Superior Agronomia, Universidade Técnica de Lisboa, Lisboa, Portugal; 3 Instituto Nacional de Investigação Agrária e Veterinária, I.P., Oeiras, Portugal; 4 Departamento Produção Vegetal, Centro de Ciências Agrárias, Universidade Federal do Espírito Santo, Alegre, Espirito Santo, Brazil; 5 Departamento Ciências da Terra, Faculdade de Ciências e Tecnologia, Universidade Nova de Lisboa, Caparica, Portugal; 6 Departamento Biologia Vegetal, Universidade Federal de Viçosa, Viçosa, Minas Gerais, Brazil; Universidade Federal de Vicosa, Brazil

## Abstract

Coffee is one of the world’s most traded agricultural products. Modeling studies have predicted that climate change will have a strong impact on the suitability of current cultivation areas, but these studies have not anticipated possible mitigating effects of the elevated atmospheric [CO_2_] because no information exists for the coffee plant. Potted plants from two genotypes of *Coffea arabica* and one of *C. canephora* were grown under controlled conditions of irradiance (800 μmol m^-2^ s^-1^), RH (75%) and 380 or 700 μL CO_2_ L^-1^ for 1 year, without water, nutrient or root development restrictions. In all genotypes, the high [CO_2_] treatment promoted opposite trends for stomatal density and size, which decreased and increased, respectively. Regardless of the genotype or the growth [CO_2_], the net rate of CO_2_ assimilation increased (34-49%) when measured at 700 than at 380 μL CO_2_ L^-1^. This result, together with the almost unchanged stomatal conductance, led to an instantaneous water use efficiency increase. The results also showed a reinforcement of photosynthetic (and respiratory) components, namely thylakoid electron transport and the activities of RuBisCo, ribulose 5-phosphate kinase, malate dehydrogenase and pyruvate kinase, what may have contributed to the enhancements in the maximum rates of electron transport, carboxylation and photosynthetic capacity under elevated [CO_2_], although these responses were genotype dependent. The photosystem II efficiency, energy driven to photochemical events, non-structural carbohydrates, photosynthetic pigment and membrane permeability did not respond to [CO_2_] supply. Some alterations in total fatty acid content and the unsaturation level of the chloroplast membranes were noted but, apparently, did not affect photosynthetic functioning. Despite some differences among the genotypes, no clear species-dependent responses to elevated [CO_2_] were observed. Overall, as no apparent sign of photosynthetic down-regulation was found, our data suggest that *Coffea* spp. plants may successfully cope with high [CO_2_] under the present experimental conditions.

## Introduction

Over the past 800,000 years, atmospheric [CO_2_] has varied between 180 µL CO_2_ L^-1^ (glacial periods) and 280 µL CO_2_ L^-1^ (interglacial periods) as Earth moved between ice ages. From pre-industrial levels of 280 µL CO_2_ L^-1^, [CO_2_] has increased steadily to 384 µL CO_2_ L^-1^ in 2009 (reached 400 µL CO_2_ L^-1^, measured in Mauna Loa Observatory in Hawaii in 2013), and levels are estimated to reach between 450 and 600 μL L^-1^ by the year 2050 and between 730 and 1020 μL L^-1^ by 2100, depending on future scenarios of anthropogenic emissions [1,2].

Changes in atmospheric [CO_2_] affect fundamental plant processes and may alter plant growth, agronomic yields and quality [3-6]. Crops sense and respond directly to rising atmospheric [CO_2_] through changes in photosynthesis and stomatal conductance (g_s_), which is the basis of what can be considered a CO_2_ fertilization effect on crop yield [7]. Such an effect may eventually strengthen the plant and alter its tolerance limits to environmental constraints. For example, it is known that high temperatures can reduce net C gain by increasing photorespiration. Additionally, a high [CO_2_] supply can increase the net photosynthetic rate (P_n_) in C_3_ plants (often above 50%) due to the higher carboxylation rate of ribulose-1,5-bisphosphate carboxylase/oxygenase (RuBisCo), which results from the simultaneous increases in substrate availability and competitive inhibition to O_2_ [3,7-10]. Therefore, by reducing photorespiration, CO_2_ enrichment is expected to enhance P_n_ to a greater degree at high than at low temperatures, thereby, at least partially, offsetting the effects of supra-optimal temperatures on yield [2,11,12].

Overall, g_s_ is consistently but not universally decreased at elevated [CO_2_] [13-15] what may decrease the transpiration rates (T_r_). Because increasing atmospheric [CO_2_] enlarges the gradient that ensures adequate diffusion of CO_2_ from the atmosphere to the chloroplasts, a rise in [CO_2_] should produce greater P_n_ coupled with lower T_r_, which would ultimately improve the water-use efficiency (WUE) in a large number of plant species [8,14,16]. Furthermore, decreases in stomatal opening, stomatal density (SD) and stomatal index (SI), all of which contributing to a reduction in g_s_, have been reported when plants are grown in elevated [CO_2_] [8,17-19], although in some cases g_s_ and not SD determines the long-term reduction in leaf T_r_ [20]. Nonetheless, the anticipated stomatal closure that is usually observed at elevated [CO_2_] will inevitably be associated with lower latent heat loss, thereby increasing leaf temperatures [2].

The degree of downregulation (acclimation) of photosynthesis in response to CO_2_ enrichment is variable among plants, depends on the interactions with other environmental limitations [7,10,21] and even changes with the developmental stage of the same plant [22]. If present, the downregulation of photosynthesis decreases (but does not completely eliminate) the stimulation effect of elevated [CO_2_] [16]. Concomitantly, CO_2_ enrichment may lead to increases in N- and water-use efficiency and decreases in leaf and plant nutrient concentrations. Yet a strong P_n_ increase under high growth [CO_2_] has often been reported to enhance relative growth rate to a much lower extent (approximately 10%). This discrepancy may result from a limitation on sink strength that prevents the plant from fully utilizing the higher photosynthate production due to, e.g., a limitation of meristematic tissue related to a deterministic growth pattern. Such a limitation could lead to an increase in leaf nonstructural carbohydrates (NSC) associated with a lower export rate to other tissues [10,23], and implicating a reduction of P_i_ regeneration in the chloroplast [5,24]. Such NSC rise could trigger a signaling mechanism, promoting a cascade of molecular and biochemical responses, e.g., the depression of gene expression and the amount/activity of photosynthetic enzymes, including that of RuBisCo, or a reduction in the levels of all components of the photosynthetic apparatus [21,23]. That would contribute to the lower the rates of net assimilation and photosynthetic capacity, linked to reductions in the maximum apparent carboxylation velocity, V_cmax_, and the *in vivo* maximum apparent rate of electron transport, J_max_ [5,7-9,19,22,25]. In fact, the reduction of photosynthesis has been mechanistically and quantitatively attributed to decreased V_cmax_ and investment in RuBisCo [13], but the responsible factors may also include reduced ribulose 1,5-bisphosphate (RuBP) regeneration, which decreases J_max_ due to lowered electron transport capacity or P_i_ availability in the chloroplast for ATP synthesis [3,8,10,21,22]. Trees and shrubs, particularly fast-growing species, generally have a larger sink capacity (root-trunk system) than annuals and usually show higher photosynthetic stimulation when grown at high [CO_2_] than do shrubs and annual crops [8,13,26].

Coffee is a tropical crop that is currently grown in approximately 80 countries, making it one of the world’s most traded agricultural products. The sale of coffee generates over US $90,000 million each year and is the economic basis of many tropical developing countries. The genus *Coffea* comprises more than 100 species, among which *C. arabica* L. and *C. canephora* Pierre ex A. Froehner that, together, are responsible for approximately 99% of world coffee bean production [27,28]. Due to the world’s ongoing climatic changes, there has been increasing concern regarding the suitability of traditional coffee producing areas. Modeling studies of predicted climate scenarios, mostly related to increased air temperatures, have estimated dramatic effects on this crop, including severe yield losses in Mexico [29], extensive reductions of suitable areas in Brazil [30] and the extinction of wild populations of *C. arabica* in Ethiopia [31]. However, these negative predictions of the effects of global climate changes have not considered the mitigating effects of increasing atmospheric [CO_2_] on the harmful impacts of elevated temperatures on the crop. Moreover, the ability that coffee plants often display to successfully adjust their metabolism to stressful environmental conditions [27,32-34] must also be taken into account.

Despite the agronomic importance of coffee, no information exists regarding the effects of CO_2_ on the physiology of this crop. The coffee plant displays low P_n_, typically in the range of 4-11 μmol m^-2^ s^-1^, at current atmospheric [CO_2_] and saturating light conditions [2], which is largely due to diffusive, rather than biochemical, limitations to photosynthesis [35,36]. It has also been proposed that coffee plants are, within given limits, capable of avoiding the downregulation of photosynthesis through their high capacity for starch accumulation [36,37], and thus we hypothesize that coffee will sustain relatively high P_n_ in a scenario of increasing atmospheric [CO_2_]. Here, we report the first results concerning the underlying mechanisms associated with the responses of the photosynthetic apparatus to elevated atmospheric [CO_2_] in coffee. We investigated three important genotypes from *C. arabica* and *C. canephora* grown at either 380 or 700 µL CO_2_ L^-1^ air under controlled conditions without water, nutrient or root development restrictions. Our results are discussed in the context of current models of photosynthetic performance in a scenario of increasing atmospheric [CO_2_].

## Materials and Methods

### Plant material and experimental design

Potted plants of 1.5 years in age from *C. arabica* L. (cv. Icatu and cv. Catucaí IPR 108) and *C. canephora* Pierre ex Froehner cv. Conilon Clone 153 (CL 153), grown in 12 L pots, were transferred from a greenhouse (ambient [CO_2_]) into walk-in growth chambers (EHHF 10000, ARALAB, Portugal). The plants were then grown under controlled environmental conditions of temperature (25/20°C, day/night), RH (75%), irradiance (approximately 800 μmol m^-2^ s^-1^), photoperiod (11.5 hours) and two CO_2_ concentrations (380 or 700 μL L^-1^). Each of the 10 plants per genotype and [CO_2_] treatment was fed on a monthly basis with 5 g of the following fertilizer mixture: 7% Ca(NO_3_)_2_, 5% KNO_3_, 7.8% P_2_O_5_, 17% K_2_O, 1.6% MgO, 20% MgSO_4_, 0,02% H_3_BO_3_ and 0.01% ZnSO_4_. To reinforce the N and Ca availability, a complementary fertilization of 2 g was conducted every 3 months with a mixture of 27% NH_4_NO_3_ and 6% CaO. Both fertilizers were provided as solid spheres that slowly dissolved over successive watering, allowing a gradual release of minerals to the soil/plant. To complement the availability of micronutrients, 500 mL of a solution containing 0.02% Fe-EDTA, 0.01% CuSO_4_, 0.01% MnCl_2_, and 0.005% H_2_MoO_2_, were added on a monthly basis.

After one year, a large set of parameters was evaluated in recently mature leaves from the top branches (light exposed) that had fully developed under each [CO_2_]. For biochemical analysis, leaf material was collected after approximately 2 hours of illumination from 6 to 8 plants of each genotype, flash frozen in liquid nitrogen and stored at -80°C until analysis. Whenever possible, all analyses were performed on the same leaves. There was no apparent restriction on root development, as judged by visual examination at the end of the experiment when plants were removed from their pots.

### Stomatal determinations and specific leaf area

As stomatal traits (e.g., stomatal density) are responsive to a range of environmental factors in addition to their relative position on the leaf [38], observations were made on similar areas of the leaf lamina by taking imprints from the abaxial leaf surface at the point of maximal leaf width, from the margin to the main central leaf vein, using colorless nail polish and adhesive transparent cellophane tape. The imprints were then placed on glass slides and examined under a light microscope (Olympus BX50, Japan) at 400x magnification, attached to a digital camera (Camera Zeiss Axiovision, Germany). Five samples per replicate per genotype, with three fields of view within each sampled area, were analyzed.

The stomatal traits were analyzed following [39]. Stomatal density (SD) was calculated as the number of stomata per leaf area unit, whereas the stomatal index (SI) was calculated as SI = [(stomata)/(total cells +stomata)] ×100. For stomatal size (SS), 30 randomly selected stomata were measured using an ocular micrometer, and their areas were calculated as SS = πab, where a and b are 1/2 length and 1/2 width, respectively, thus assuming that the stomatal shape is an ellipse.

The specific leaf area (SLA) was determined in 5 samples of 10 leaf discs (0.5 cm^-2^ each) after drying at 80°C for 24 h.

### Gas exchanges

The responses of the net photosynthetic rate (P_n_) to internal CO_2_ concentration (C_i_) (P_n_/C_i_ curves) were assessed using a portable open-system infrared gas analyzer (Li-Cor 6400, LiCor, Lincoln, USA). The curves were obtained from 5 to 7 plants per genotype under the light conditions of the growth chambers (*ca*. 800 µmol m^-2^ s^-1^) using a 10-12 stepwise external [CO_2_] levels from 50 to 1800 μL L^-1^. Each step value was taken only when P_n_, stomatal conductance to water vapor (g_s_) and transpiration rate (T_r_) were stable, which was approximately 5-7 min after each [CO_2_] level was imposed. From the P_n_/C_i_ curves, were estimated the *in vivo* maximum apparent rate of carboxylation (V_cmax_), the *in vivo* maximum apparent rate of electron transport (J_max_), the respiration in the presence of light (R_d_) and the triose phosphate utilization rate (TPU), as described elsewhere [40,41]. As the conditions inside the growth chamber were quite stable (including air humidity kept at 75%) and no limitations (e.g., water, nutrients) were imposed, the leaf temperature was no higher than 1 °C above air temperature (25 °C). Therefore, in the experimental conditions, the leaf-to-air vapor pressure deficit was also quite constant and low (the highest value was close to 0.985 kPa).

The steady-state values of leaf P_n_, g_s_ and T_r_ were taken from the same P_n_/C_i_ curves at 380 or 700 µL CO_2_ L^-1^. Leaf instantaneous water-use efficiency (iWUE) was calculated as the P_n_-to-T_r_ ratio, representing the units of assimilated CO_2_ per unit of water lost through transpiration.

The photosynthetic capacity, A_max_ (representing the light- and CO_2_-saturated rate of photosynthesis under optimal temperature), was measured through O_2_ evolution in a Clark-type O_2_ electrode (LD2/2, Hansatech, UK) using leaf discs (1.86 cm^2^), following the procedures described elsewhere [42]. A_max_ was obtained at 25 °C under saturating [CO_2_] conditions (*ca*. 7%, supplied by 400 μl KHCO_3_, 2 M) by exposing the leaf samples to increasing irradiances up to 1500 μmol m^-2^ s^-1^ using a Björkman lamp (Hansatech) and neutral filters.

### Chlorophyll a fluorescence analysis

Chlorophyll (Chl) *a* fluorescence parameters were determined on the same leaves used for the gas exchange measurements using a PAM-2000 system (H. Walz, Effeltrich, Germany), as previously described [42], following formulae discussed elsewhere [43,44]. Briefly, the estimation of the maximal photochemical efficiency of photosystem (PS) II (F_v_/F_m_) was performed on overnight dark-adapted leaves, using a 0.8 s saturating light pulse of 7500 µmol m^-2^ s^-1^. The photochemical quenching (q_P_), which denotes the proportion of energy trapped by PSII and driven to photochemical events, and the maximal PSII efficiency of energy conversion under light (F_v_´/F_m_´) were determined under photosynthetic steady-state conditions, at the light conditions of the growth chambers, using superimposed saturating flashes.

### Thylakoid electron transport rates

Subchloroplast fractions were obtained from a pool of leaf material (*ca*. 5 g FW) from 5 or 6 plants and processed as previously optimized for coffee leaves [33,45]. The *in vivo* electron transport rates associated with both PSI (DCPIPH_2_→MV) and PSII, including (H_2_O→DCPIP) or excluding (DPC→DCPIP) the oxygen evolving complex (OEC), were measured polarographically using an LW2 O_2_ electrode (Hansatech) at 25 °C. The assays were performed using 1 mL of the reaction mixture (containing *ca*. 100 mg Chl) and a PPFD of approximately 3000 µmol m^-2^ s^-1^ given by a Björkman lamp (Hansatech).

### Enzyme activities

Four freshly cut leaf discs (0.5 cm^2^ each) were used to measure the activity of several enzymes involved in carbon metabolism. Each sample was homogenized in a cooled mortar using 100 mg insoluble PVPP and 1 mL of the extraction buffer 100 mM Tris-HCl (pH 8), which contained 10 mM MgCl_2_, 10 mM NaHCO_3_, 10 mM ß-mercaptoethanol, 2 mM DTT, 1% (v/v) Triton X-100, 10% (v/v) glycerol and a “complete-protease inhibitor cocktail” 2% (v/v) designed to protect the enzymes from protease action (Roche, ref. 04693159001). The extracts were centrifuged (16,000 *g*, 20 min, 4 °C) and the supernatant was used for the enzyme assays, all of which were based on NADH oxidation at 340 nm, at 25 °C, in 1 mL final volume in the cuvette.

The total ribulose-1,5-bisphosphate carboxylase/oxygenase (RuBisCo: EC 4.1.1.39) activity was determined following previously described procedures [46], with some modifications: 20 μL of the leaf extract were added to a cuvette containing an assay medium of 50 mM Tris-HCl, pH 8.0, 15 mM MgCl_2_, 20 mM NaHCO_3_, 100 mM creatine phosphate, 10 mM ATP, 0.2 mM NADH, 20 U mL^-1^ creatine kinase, 15 U mL^-1^ 3-phosphoglycerate kinase and 15 U mL^-1^ glyceraldehyde-3-phosphate dehydrogenase. After an incubation period of 10 min, 50 μL of 20 mM RuBP were added to initiate the reaction.

The activity of ribulose 5-phosphate kinase (RuB5PK: EC 2.7.1.19) was determined following procedures described elsewhere [41], by adding 20 μL of the leaf extract to a cuvette containing an assay medium of 100 mM Tris-HCl, pH 8.0, 8 mM MgCl_2_, 40 mM KCl, 20 mM phosphoenolpyruvate, 5 mM ATP, 1 mM NADH, 20 mM DTT, 8 units pyruvate kinase, 10 U mL^-1^ lactate dehydrogenase and 5 U mL^-1^ phosphoriboisomerase. After an incubation period of 15 min, the reaction was initiated by adding 10 μL of 500 mM ribose-5-phosphate.

The pyruvate kinase (PK: EC 2.7.1.40) activity was determined as previously described [47], with some changes: 20 μL of the leaf extract were added to a cuvette containing an assay medium of 100 mM Tris-HCl (pH 7.0), 10 mM MgCl_2_, 0.2 mM NADH, 1 mM fructose-1,6-bisphosphate, 45 mM ADP and 6.3 U mL^-1^ lactate dehydrogenase. The reaction was initiated by adding 100 μL of 10 mM phosphoenolpyruvate.

The determination of NADH-dependent malate dehydrogenase (MDH: EC 1.1.1.37) activity followed procedures described elsewhere [48], with some changes, by adding 20 μL of the leaf extract to a cuvette containing an assay medium of 50 mM Tris-HCl (pH 8.0) and 0.1 mM NADH. The reaction was initiated by adding 20 μL of 20 mM oxaloacetate as substrate.

### Non-structural carbohydrate quantification

Soluble sugars were determined in approximately 150 mg of powdered frozen material, based on the method previously described [49]. The samples were homogenized in 2 mL of cold H_2_O, left to extract for 20 min on ice and centrifuged (12,000 *g*, 5 min, 4 °C). The supernatant was boiled to denature the proteins (3 min), placed on ice (6 min) and centrifuged again. The obtained clear solution was then filtered (0.45 µm, nylon) before the injection of a 50 μL aliquot into an HPLC system equipped with a refractive index detector (Model 2414, Waters, USA). The separation of sugars was performed using a Sugar-Pak 1 column (300 x 6.5 mm, Waters) at 90 °C, with H_2_O as the eluent (containing 50 mg EDTA-Ca L^-1^ H_2_O) and a flow rate of 0.5 mL min^-1^. To overcome the presence of non-pure peaks from this separation, another 20 μL aliquot of each sample was injected through a DionexCarboPac PA1 analytical column (4 x 250 mm, Thermo Scientific, USA) coupled to a DionexCarboPac PA1 Guard (4 × 50 mm) at 20 °C. Ultrapure water and 300 mM NaOH were used as eluents (water from 0 to 50 min; NaOH from 50 to 65 min; and water from 65 to 80 min for re-equilibration) at a 1 mL min^-1^ flow rate. Standard curves were used for the quantification of each sugar.

Starch determination was conducted according to previously described procedures [50], with some changes. After adding 1 mL of boiling millipore water to 100 mg of frozen leaf material, the samples were immediately placed into boiling water, shaken for 10 min and centrifuged (10,000 *g*, 2 min, 4°C). The supernatant containing the soluble sugars was discarded, and the insoluble pellet containing starch was collected, further washed and centrifuged twice in 1 mL millipore water. The insoluble residue was thoroughly homogenized in 1 mL H_2_O, and the resulting suspension was autoclaved (120°C, 3 h) to promote starch gelatinization. Starch hydrolysis was performed in 0.5 mL of the solution by adding 0.5 mL of 400 mM citrate/KOH buffer, pH 4.6, containing 30 U amyloglucosidase (EC 3.2.1.3, Sigma) and 2 U α-amylase (EC 3.2.1.1, Sigma), followed by an overnight shaking incubation at 37 °C. The extract was then cleared by centrifugation (10,000 *g*, 2 min, 4°C), and the glucose derived from starch was enzymatically determined on a 20 μL aliquot of the supernatant, using 100 mM imidazol buffer, pH 6.9, containing 1.1 mM ATP, 0.5 mM NADP, 1 U hexokinase (EC 2.7.1.1) and 1 U glucose-6-phosphate dehydrogenase (EC 1.1.1.49) in a 1 mL final volume. The determination was conducted spectrophotometrically at 340 nm.

### Photosynthetic pigments

Total chlorophylls and carotenoids were extracted from four freshly cut leaf discs (0.5 cm^2^ each) using 80% (v/v) aqueous acetone and quantified according to procedures described elsewhere [51].

### Membrane permeability

Ten freshly cut leaf discs (0.5 cm^2^ each) were rinsed 3 times (1 min) with demineralized water and subsequently floated on 10 mL of demineralized water at 20°C, following previously described procedures [52]. The electrolyte leakage was measured until leakage stabilization occurred, at 22 h, using a conductivity meter (Crison GLP31, Crison Instruments, S.A., Spain). Total conductivity was obtained after the flasks were exposed to 90°C for 2 h in an oven and cooled. Membrane leakage was given as a percentage of total conductivity.

### Lipid quantification from chloroplast membranes

The lipid fraction from the enriched chloroplast membranes was obtained from 3 to 4 g (FW) of leaf tissue, followed by the quantification and identification of fatty acids (FAs), as has been previously described for coffee leaves [28]. The value for the total fatty acid (TFA) content corresponds to the sum of individual FAs, while the double bond index (DBI) was calculated as DBI = [(% monoenes + 2 x % dienes + 3 x % trienes / (% saturated FAs)].

### Statistical analysis

The various measured and calculated parameters were analyzed using two-way ANOVAs (P < 0.05) to evaluate the differences between CO_2_ treatments or genotypes, followed by a Tukey test for mean comparisons among genotypes within the same [CO_2_] condition and an F test for mean comparisons between [CO_2_] treatments within the same genotype; a 95% confidence level was adopted for all tests. For the sake of simplicity concerning the ANOVA results, in the figure and table captions for each parameter are only indicated when significant the differences related to growth [CO_2_], genotype of for the [CO_2_] x genotype interaction.

## Results

### Stomatal traits and specific leaf area

Preliminary observations confirmed that coffee plants have stomata only on the abaxial leaf surface. A consistent trend for lower stomatal density, SD (5-14%), and higher stomatal size, SS (3-7%), was found under high [CO_2_] in all genotypes, although statistical significance was reached only in the genotype Icatu (Table 1). Regardless of genotype, the stomatal index (SI) and specific leaf area (SLA) did not respond significantly to the [CO_2_] treatments.

**Table 1:** The stomatal density (SD), stomatal size (SS), stomatal index (SI) and specific leaf area (SLA) in the leaves of *C. arabica* (Icatu and IPR 108) and *C. canephora* (Conilon CL 153) grown under 380 or 700 μL CO_2_ L^-1^.

**Genotype**	**CL 153**		**Icatu**		**IPR 108**	
**CO_2_ Treatment**	**380 μL L^-1^**	**700 μL L^-1^**	**380 μL L^-1^**	**700 μL L^-1^**	**380 μL L^-1^**	**700 μL L^-1^**
**Stomatal Density**	**195.2 ^a r^**	**174.8 ^a r^**	**193.9 ^a r^**	**165.9 ^a s^**	**190.7 ^a r^**	**180.6 ^a r^**
**(n. stomata mm^-2^)**	**8.8**	**6.8**	**8.4**	**7.2**	**4.7**	**3.6**
**Stomatal Index**	**14.0 ^b r^**	**14.8 ^b r^**	**24.0 ^a r^**	**20.7 ^a r^**	**26.2 ^a r^**	**22.6 ^a r^**
**(%)**	**0.4**	**0.4**	**1.1**	**0.8**	**1.3**	**0.5**
**Stomatal Size**	**273.2 ^c r^**	**289.8 ^c r^**	**349.7 ^b s^**	**374.8 ^b r^**	**398.1 ^a r^**	**409.3 ^a r^**
**(μm^2^)**	**7.7**	**7.5**	**7.5**	**7.3**	**6.1**	**9.1**
**SLA**	**13.5 ^a r^**	**13.6 ^a r^**	**13.4 ^a r^**	**11.6 ^a r^**	**15.8 ^a r^**	**12.9 ^a r^**
**(m^2^ Kg^-1^)**	**2.2**	**3.8**	**1.4**	**1.2**	**1.8**	**1.4**

For each parameter, the mean values ± SE (n = 30 for SD, SS and SI; n = 5 for SLA) followed by different letters express significant differences between cultivars for the same CO_2_ treatment (a, b, c) or between CO_2_ treatments within the same cultivar (r, s). The ANOVA for SD showed significant differences only between CO_2_ treatments within the same cultivar; that for SI showed significant differences between cultivars for the same CO_2_ treatment; and that for SS showed significant differences between cultivars for the same CO_2_ treatment and between CO_2_ treatments within the same cultivar; that for SLA did not show significant differences.

### Leaf gas exchanges

Irrespective of genotype, a tendency to higher values was observed for the net photosynthetic rate (P_n_) under higher [CO_2_] (between 34% in IPR 108 and 49% in CL 153). Furthermore, P_n_ measured at 380 or 700 μL CO_2_ L^-1^ showed similar values in the plants grown either at normal or elevated [CO_2_], thus the changes in P_n_ were independent of growth [CO_2_] conditions (data not shown).

**Figure 1:**
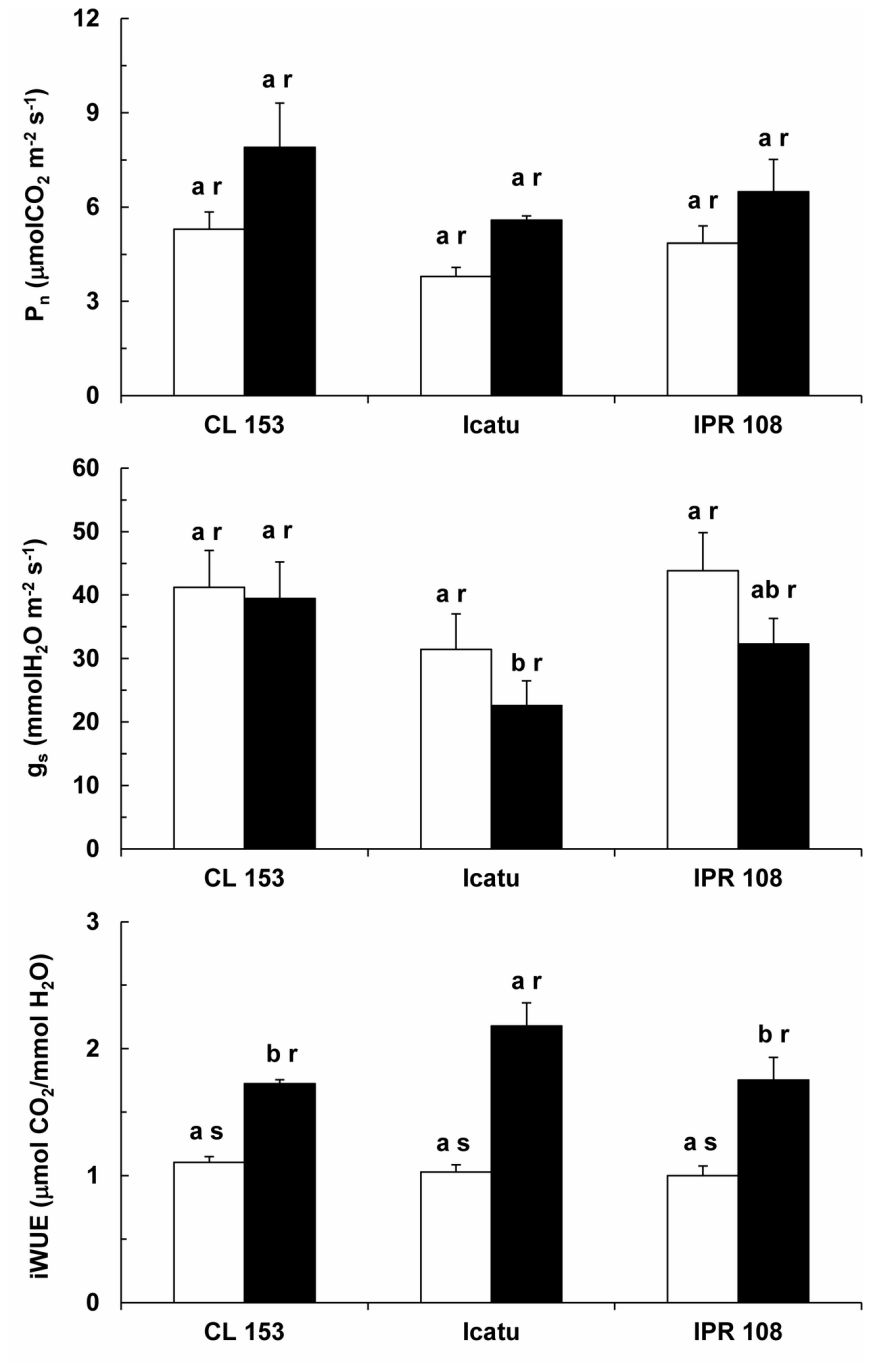
Gas exchanges under ambient [CO_2_]. Changes in leaf net photosynthesis (P_n_), stomatal conductance to water vapor (g_s_) and instantaneous water use efficiency (iWUE) in *C. arabica* (Icatu and IPR 108) and *C. canephora* (Conilon CL 153) measured at their growth [CO_2_]: 380 (white bars) and 700 (black bars) μL CO_2_ L^-1^. For each parameter, the mean values + SE (n = 6-8) followed by different letters express significant differences between cultivars for the same CO_2_ treatment (a, b) or between CO_2_ treatments within the same cultivar (r, s). The ANOVA for P_n_, showed significant differences between cultivars for the same growth CO_2_ treatment and between CO_2_ treatments within the same cultivar; that for iWUE showed significant differences between CO_2_ treatments within the same cultivar; that for g_s_ did not show any significant difference,.

Concerning the stomatal conductance to water vapor (g_s_), no significant changes were observed between the [CO_2_] treatments for each genotype. However, g_s_ values tended to be lower when measured at 700 μL CO_2_ L^-1^, from 4% (CL 153) to 28% (Icatu) (Figure 1). Again, the non-significant decreasing trend observed in g_s_ when measured at 700 μL CO_2_ L^-1^ was irrespective of growth [CO_2_] (data not shown).

Despite the absence of significant changes in the above parameters when measured at 700 μL CO_2_ L^-1^ in comparison to 380 μL CO_2_ L^-1^, the opposite tendencies of g_s_ (and T_r_) and P_n_ when measured at high [CO_2_] led to significant increases in the instantaneous water use efficiency (iWUE) in all genotypes, between 56% and 112% (Figure 1).

**Figure 2:**
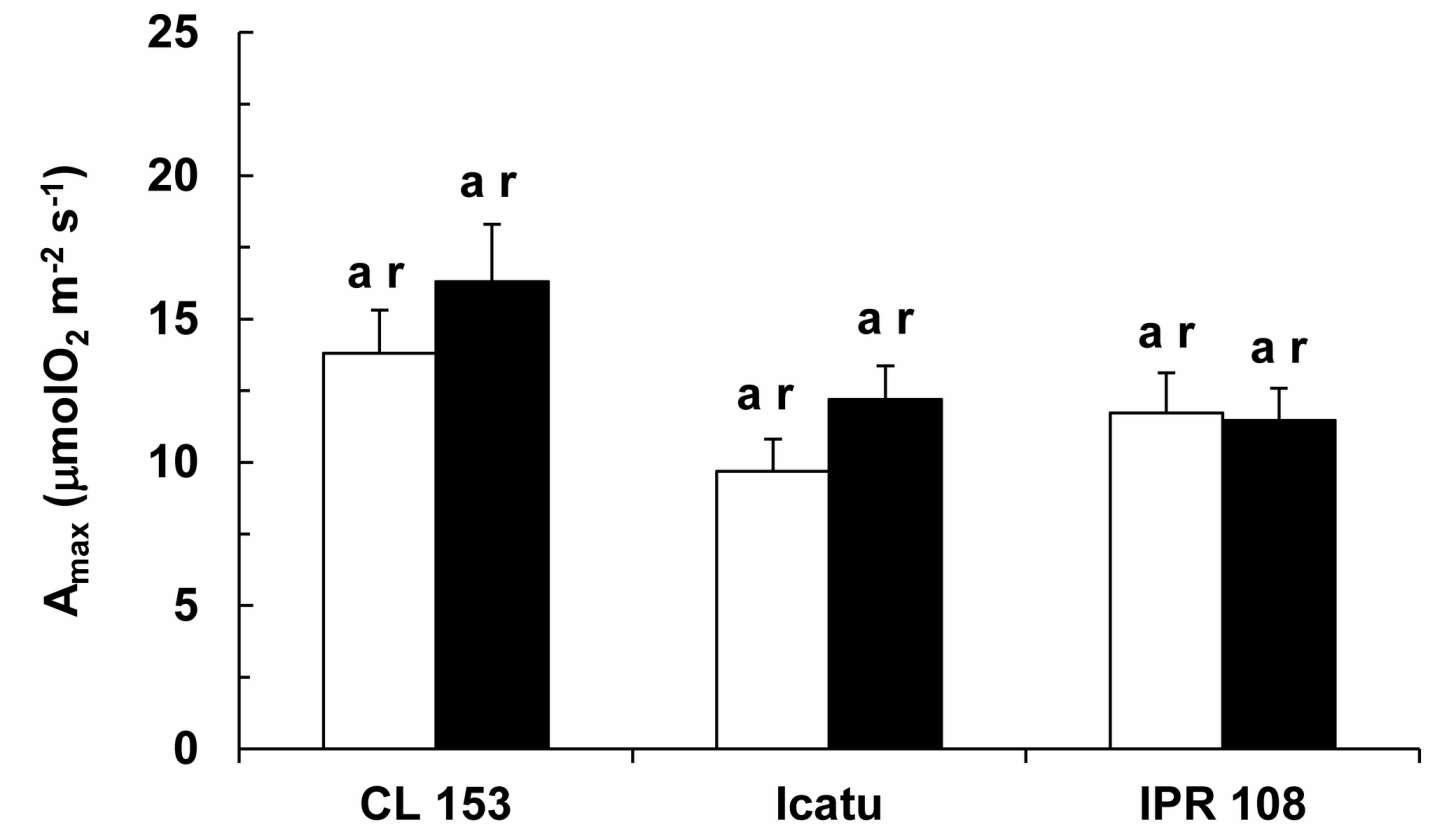
Potential photosynthesis. Changes in photosynthetic capacity (A_max_) in the leaves of *C. arabica* (Icatu and IPR 108) and *C. canephora* (Conilon CL 153) grown under 380 (white bar) and 700 (black bar) μL CO_2_ L^-1^. The mean values + SE (n = 6) followed by different letters express significant differences between cultivars for the same CO_2_ treatment (a, b) or between CO_2_ treatments within the same cultivar (r, s). The ANOVA for A_max_ showed significant differences between cultivars for the same growth CO_2_ treatment.

Among the genotypes, differences in the functioning of the photosynthetic apparatus were found. Both the *in vivo* apparent rates for the maximum carboxylation (V_cmax_) and maximum electron transport (J_max_) were unresponsive to CO_2_ enrichment in CL 153 and IPR 108, whereas in Icatu these parameters increased 52% and 37%, respectively, at elevated relative to normal growth [CO_2_] (Table 2). The balance between RuBisCo carboxylation and electron transport (V_cmax_/J_max_ ratio) was unaltered by growth [CO_2_], averaging 1.21 across all genotypes. Similarly, the triose phosphate utilization for sucrose and starch synthesis (TPU), dark respiration rate, R_d_ (Table 2) and photosynthetic capacity, A_max_ (Figure 2), were also unresponsive to the applied CO_2_ treatments, although A_max_ tended to increase with CO_2_ in CL 153 (18%) and Icatu (25%).

**Table 2:** Variation of the estimations of the maximum rate of carboxylation (V_cmax_), the maximum rate of carboxylation limited by electron transport (J_max_), the rate of respiration in the presence of light (R_d_), and the triose-phosphate (TPU) rate of utilization (all calculated from the P_n_/Ci curves and expressed in μmol CO_2_ m^-2^ s^-1^), as well as the J_max_/V_cmax_ ratio, in the leaves of *C. arabica* (Icatu and IPR 108) and *C. canephora* (Conilon CL 153) grown under 380 and 700 μL L-1 of CO_2_.

**Genotype**	**CL 153**		**Icatu**		**IPR 108**	
**CO_2_ Treatment**	**380 μL L^-1^**	**700 μL L^-1^**	**380 μL L^-1^**	**700 μL L^-1^**	**380 μL L^-1^**	**700 μL L^-1^**
**V_cmax_**	**50.2 ^a r^**	**48.5 ^a r^**	**34.8 ^b s^**	**53.0 ^a r^**	**43.7 ^a r^**	**51.8 ^a r^**
**(μmol CO_2_ m^-2^ s^-1^)**	**4.5**	**2.1**	**2.2**	**8.6**	**2.1**	**5.8**
**J_max_**	**58.6 ^a r^**	**62.0 ^a r^**	**45.1 ^b s^**	**62.0 ^a r^**	**52.7 ^ab r^**	**53.4 ^a r^**
**(μmol CO_2_ m^-2^ s^-1^)**	**2.1**	**8.5**	**2.6**	**6.7**	**2.5**	**6.0**
**J_max_/V_cmax_**	**1.25 ^a r^**	**1.25 ^a r^**	**1.31 ^a r^**	**1.25 ^a r^**	**1.22 ^a r^**	**1.12 ^a r^**
	**0.15**	**0.09**	**0.10**	**0.10**	**0.06**	**0.10**
**R_d_**	**2.11 ^a r^**	**2.38 ^a r^**	**1.74 ^a r^**	**3.68 ^a r^**	**1.80 ^a r^**	**3.14 ^a r^**
**(μmol CO_2_ m^-2^ s^-1^)**	**0.76**	**0.45**	**0.46**	**0.54**	**0.41**	**0.61**
**TPU**	**3.19 ^a r^**	**3.51 ^a r^**	**2.91 ^a r^**	**3.68 ^a r^**	**3.26 ^a r^**	**3.41 ^a r^**
**(μmol CO_2_ m^-2^ s^-1^)**	**0.17**	**0.25**	**0.11**	**0.27**	**0.13**	**0.35**

For each parameter, the mean values ± SE (n = 5-8) followed by different letters express significant differences between cultivars for the same CO_2_ treatment (a, b) or between CO_2_ treatments within the same cultivar (r, s). The ANOVAs for V_cmax_ and J_max_ showed significant differences between cultivars for the same CO_2_ treatment and between CO_2_ treatments within the same cultivar. Only for the first variable was also detected a significant interaction between genotype and growth CO_2_.

### Chlorophyll a fluorescence analysis

Only modest changes were noted for the maximal photochemical efficiency of PSII (F_v_/F_m_) in the plants grown under high [CO_2_], with a maximal reduction of 3% in Icatu (Table 3). These values were not far from those expected under shade conditions (0.78), where PSI makes a larger contribution to the F_0_ level [53]. Regardless of CO_2_ treatment, no significant changes were found under photosynthetic steady-state conditions for either photochemical quenching (q_P_) or the photochemical efficiency of PS II (F_v_´/F_m_´).

**Table 3:** Evaluation of several fluorescence parameters determined under dark adapted (F_v_/F_m_) and under photosynthetic steady-state (q_P_, F_v_´/F_m_´) conditions in the leaves of *C. arabica* (Icatu and IPR 108) and *C. canephora* (Conilon CL 153) grown under 380 and 700 μLCO_2_ L^-1^.

**Genotype**	**CL 153**		**Icatu**		**IPR 108**	
**Growth [CO_2_]**	**380 μL L^-1^**	**700 μL L^-1^**	**380 μL L^-1^**	**700 μL L^-1^**	**380 μL L^-1^**	**700 μL L^-1^**
**F_v_/F_m_**	**0.776 ^a r^**	**0.760 ^a r^**	**0.754 ^b r^**	**0.732 ^b s^**	**0.758 ^b r^**	**0.748 ^ab r^**
	**0.006**	**0.007**	**0.006**	**0.006**	**0.004**	**0.005**
**q_P_**	**0.682 ^a r^**	**0.558 ^a r^**	**0.659 ^a r^**	**0.572 ^a r^**	**0.590 ^a r^**	**0.621 ^a r^**
	**0.022**	**0.026**	**0.060**	**0.069**	**0.024**	**0.034**
**F_v_'/F_m_'**	**0.575 ^a r^**	**0.577 ^ab r^**	**0.589 ^a r^**	**0.664 ^a r^**	**0.531 ^a r^**	**0.542 ^b r^**
	**0.015**	**0.024**	**0.028**	**0.024**	**0.026**	**0.023**

For each parameter, the mean values ± SE (n = 5) followed by different letters express significant differences between cultivars for the same CO_2_ treatment (a, b) or between CO_2_ treatments within the same cultivar (r, s). Only the ANOVA for q_P_ showed significant effects between CO_2_ treatments within the same cultivar.

### Thylakoid electron transport rates

**Figure 3:**
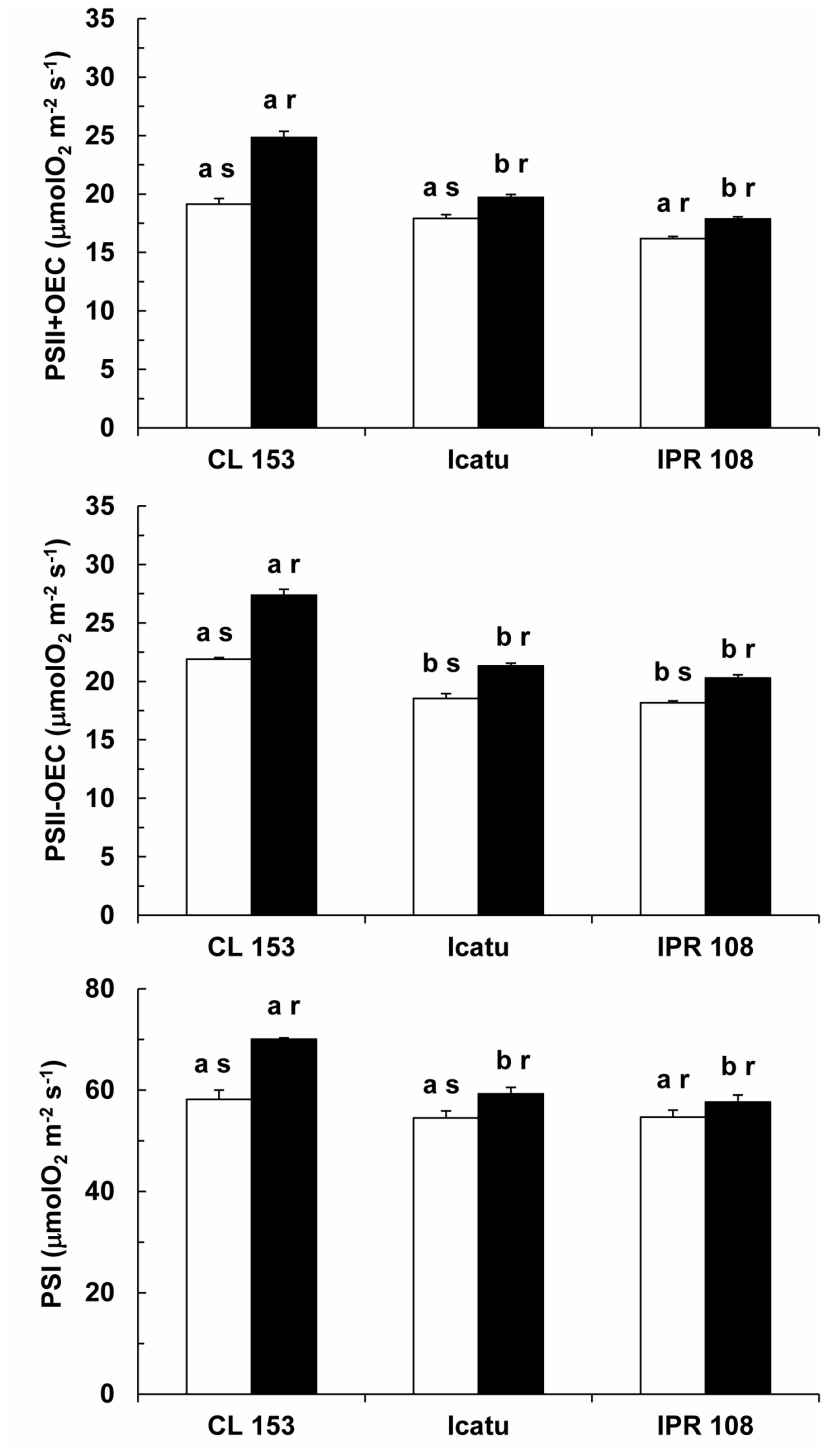
Thylakoid electron transport potential. Changes in the thylakoid electron transport rates associated with PSII (with and without the inclusion of OEC) and PSI in the leaves of *C. arabica* (Icatu and IPR 108) and *C. canephora* (Conilon CL 153) grown under 380 (white bar) and 700 (black bar) μL CO_2_ L^-1^. For each parameter, the mean values + SE (n = 4) followed by different letters express significant differences between cultivars for the same CO_2_ treatment (a, b) or between CO_2_ treatments within the same cultivar (r, s). The ANOVAs for PSII+OEC, PSII-OEC and PSI showed significant differences between cultivars for the same CO_2_ treatment and between CO_2_ treatments within the same cultivar, as well for the interaction between genotype and growth CO_2_ (except in PSI for the interaction).

The potential thylakoid electron transport rate for both photosystems changed with growth [CO_2_], although with different extent among the genotypes (Figure 3). Increases in the electron transport of photosystem II including the oxygen evolving complex (PSII+OEC) under high [CO_2_] ranged from 10% (*C. arabica* genotypes, although this increase was not significant in IPR 108) to 30% (CL 153). The PSII activity excluding the OEC (PSII-OEC) also showed significant increases, ranging from 12% (IPR 108) to 25% (CL 153). The pattern of PSI activity closely followed that of PSII, with significant enhancements of 20% in CL 153 and 9% in Icatu, whereas IPR 108 showed a non-significant rise of 6% in PSI activity.

### Enzyme activities

**Figure 4:**
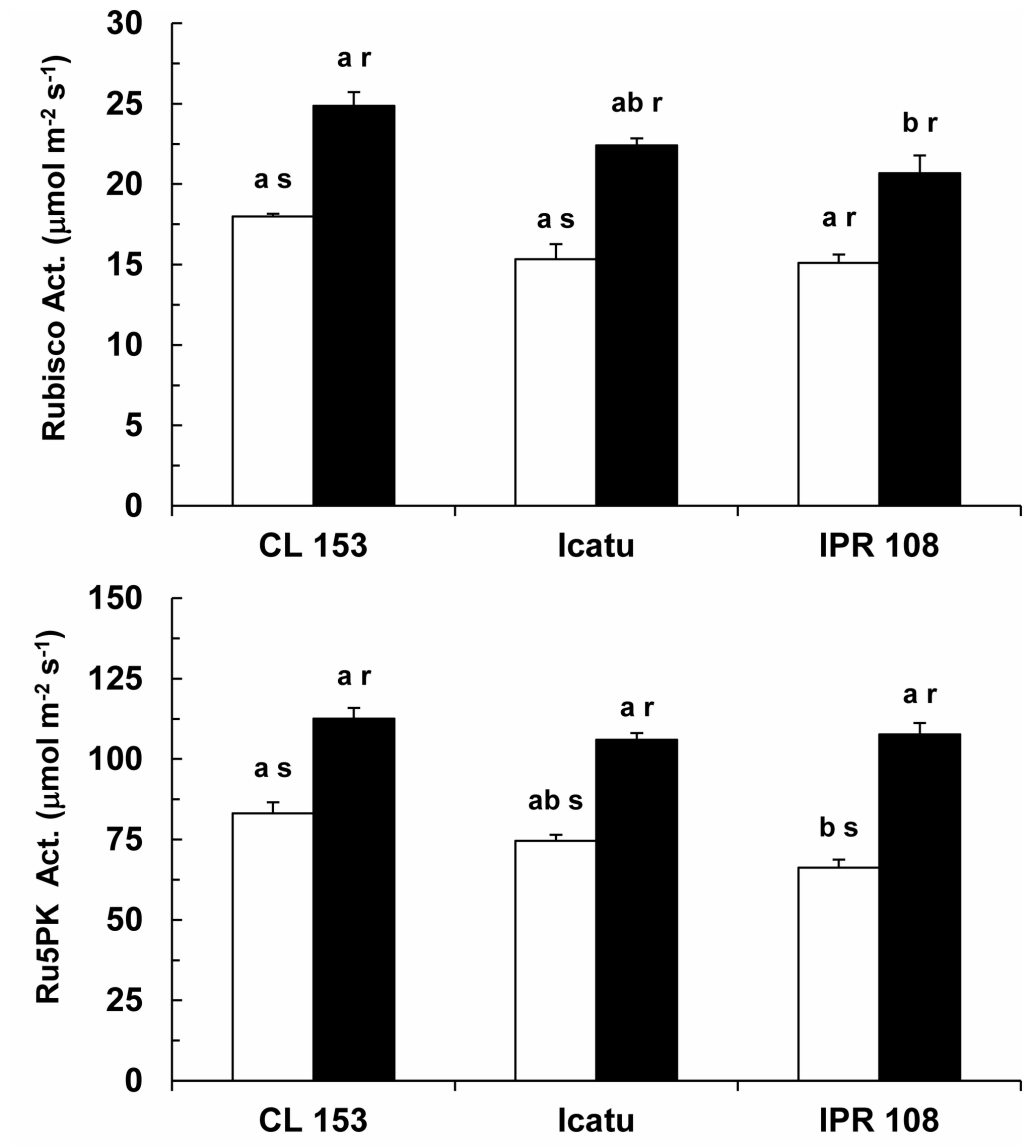
Maximal activities of some photosynthetic enzymes. Variation of the total activities of ribulose-1,5-bisphosphate carboxylase/oxygenase (RuBisCo) and ribulose 5-phosphate kinase (Ru5PK) in the leaves of *C. arabica* (Icatu and IPR 108) and *C. canephora* (Conilon CL 153) grown under 380 (white bar) and 700 (black bar) μL CO_2_ L^-1^. For each enzyme, the mean values + SE (n = 4) followed by different letters express significant differences between cultivars for the same CO_2_ treatment (a, b) or between CO_2_ treatments within the same cultivar (r, s). The ANOVA for RuBisCo showed significant differences only between CO_2_ treatments within the same cultivar, whereas that for Ru5PK showed significant differences between cultivars for the same CO_2_ treatment and between CO_2_ treatments within the same cultivar.

**Figure 5:**
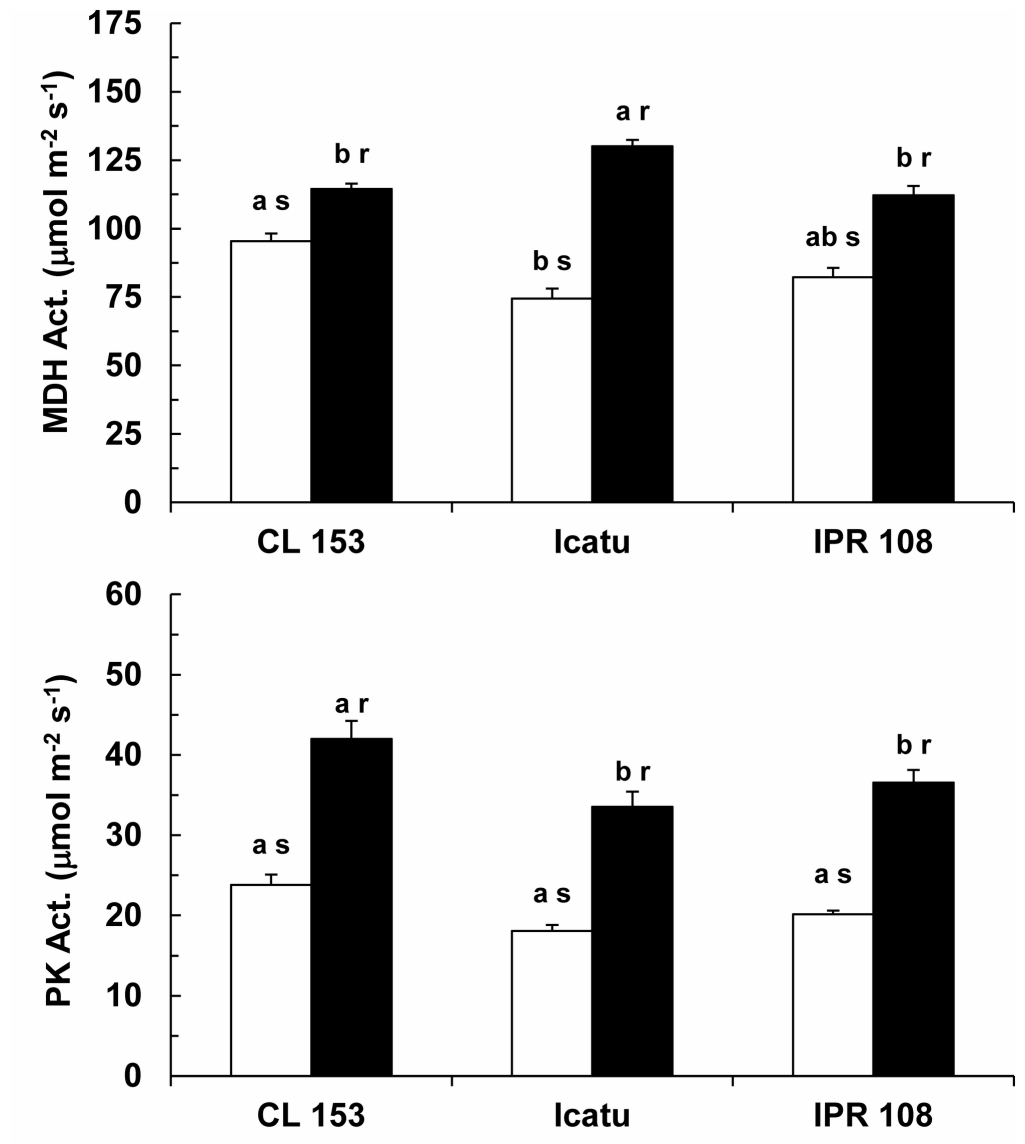
Maximal activities of some respiratory enzymes. Variation of the total activities of malate dehydrogenase (MDH) and pyruvate kinase (PK) in the leaves of *C. arabica* (Icatu and IPR 108) and *C. canephora* (Conilon CL 153) grown under 380 (white bar) and 700 (black bar) μL CO_2_ L^-1^. For each enzyme, the mean values + SE (n = 4) followed by different letters express significant differences between cultivars for the same CO_2_ treatment (a, b) or between CO_2_ treatments within the same cultivar (r, s). The ANOVAs for MDH and PK showed significant differences between the cultivars for the same CO_2_ treatment and between CO_2_ treatments within the same cultivar.

The enzyme activities were clearly affected by growth [CO_2_] in all genotypes. The potential activities of the photosynthetic related enzymes ribulose-1,5-bisphosphate carboxylase/oxygenase (RuBisCo) and ribulose 5-phosphate kinase (Ru5PK) were similar among genotypes at normal [CO_2_]. Additionally, significant increases were observed for high growth [CO_2_] plants, from 37% (IPR 108) to 46% (Icatu) for RuBisCo and from 35% (CL 153) to 63% (IPR 108) for Ru5PK (Figure 4). Similar patterns were exhibited by the key enzymes of the respiratory pathway, malate dehydrogenase (MDH) and pyruvate kinase (PK) (Figure 5), with increases in the plants grown under high [CO_2_] ranging from 20% (CL 153) to 75% (Icatu) for MDH and from 76% (CL 153) to 86% (Icatu) for PK.

### Non-structural carbohydrate quantification

Several soluble sugars showed different variation patterns under high growth [CO_2_] (Table 4). The most abundant sugar, sucrose, increased as much as 20% in IPR 108, whereas levels were more stable in CL 153 and Icatu. Glucose did not change significantly in CL 153 and Icatu but decreased by 42% in IPR 108, whereas fructose was stable in Icatu and decreased in the other two genotypes. Raffinose did not change significantly in any genotype, and arabinose decreased only in CL 153 (32%), in response to higher growth [CO_2_]. Finally, strong reductions under high growth [CO_2_] were found for trehalose (between 78 and 92%), stachyose (63-88%), galactose (58-89%) and mannitol (51-77%). Taken together, these changes led to decreases of total soluble sugar content in all genotypes, between 15% (Icatu) and 33% (CL 153).

**Table 4:** The concentrations of non-structural carbohydrates (soluble sugars and starch) in the leaves of *C. arabica* (Icatu and IPR 108) and *C. canephora* (Conilon CL 153) grown under 380 or 700 μL CO_2_ L^-1^.

**Genotype**	**CL 153**		**Icatu**		**IPR 108**	
**CO_2_ Treatment**	**380 μL L^-1^**	**700 μL L^-1^**	**380 μL L^-1^**	**700 μL L^-1^**	**380 μL L^-1^**	**700 μL L^-1^**
**Stachyose (mg g^-1^_DW_)**	**1.35 ^b r^**	**0.28 ^ab s^**	**1.11 ^c r^**	**0.41 ^a s^**	**1.60 ^a r^**	**0.20 ^b r^**
	**0.04**	**0.02**	**0.08**	**0.01**	**0.03**	**0.01**
**Raffinose (mg g^-1^_DW_)**	**3.54 ^c r^**	**3.65 ^b r^**	**4.06 ^b r^**	**3.62 ^b r^**	**4.98 ^a r^**	**5.11 ^a r^**
	**0.12**	**0.12**	**0.13**	**0.18**	**0.08**	**0.10**
**Trehalose (mg g^-1^_DW_)**	**8.12 ^ab r^**	**1.09 ^ab s^**	**7.61 ^b r^**	**1.69 ^a s^**	**8.64 ^a r^**	**0.73 ^b s^**
	**0.24**	**0.08**	**0.54**	**0.03**	**0.15**	**0.04**
**Sucrose (mg g^-1^_DW_)**	**38.76 ^b r^**	**37.67 ^b r^**	**44.12 ^a r^**	**41.52 ^a r^**	**32.12 ^c s^**	**38.70 ^ab r^**
	**1.06**	**0.35**	**0.78**	**1.68**	**0.93**	**0.39**
**Glucose (mg g^-1^_DW_)**	**4.69 ^c r^**	**5.23 ^c r^**	**16.87 ^b r^**	**16.92 ^a r^**	**25.53 ^a r^**	**14.80 ^a s^**
	**0.24**	**0.19**	**0.40**	**0.44**	**1.01**	**0.40**
**Fructose (mg g^-1^_DW_)**	**23.31 ^bc r^**	**11.47 ^b s^**	**21.57 ^c r^**	**19.91 ^a r^**	**26.93 ^a r^**	**17.60 ^a s^**
	**0.45**	**0.38**	**0.59**	**1.45**	**1.53**	**0.40**
**Galactose (mg g^-1^_DW_)**	**0.47 ^b r^**	**0.05 ^b s^**	**0.38 ^c r^**	**0.16 ^a s^**	**0.72 ^a r^**	**0.12 ^a s^**
	**0.01**	**0.00**	**0.03**	**0.00**	**0.01**	**0.01**
**Arabinose (mg g^-1^_DW_)**	**4.74 ^a r^**	**3.19 ^a s^**	**4.38 ^ab r^**	**3.88 ^a r^**	**3.76 ^b r^**	**3.13 ^a r^**
	**0.07**	**0.28**	**0.06**	**0.34**	**0.31**	**0.24**
**Mannitol (mg g^-1^_DW_)**	**11.23 ^a r^**	**2.58 ^b s^**	**9.67 ^b r^**	**4.73 ^a s^**	**10.70 ^ab r^**	**2.59 ^b s^**
	**0.33**	**0.20**	**0.69**	**0.08**	**0.19**	**0.16**
**Total Soluble (mg g^-1^_DW_)**	**96.20 ^b r^**	**64.86 ^c s^**	**109.77 ^a r^**	**92.83 ^a s^**	**114.99 ^a r^**	**82.98 ^b s^**
	**1.60**	**0.54**	**2.57**	**0.75**	**2.83**	**0.36**
**Starch (mg _gluc. equivalents_ g^-1^_DW_)**	**33.16 ^b s^**	**56.32 ^a r^**	**54.68 ^a r^**	**49.21 ^a r^**	**25.09 ^b r^**	**21.44 ^b r^**
	**1.80**	**5.73**	**1.53**	**5.07**	**0.81**	**4.20**
Total Soluble/Starch (g g^-1^) *	**2.90**	**1.15**	**2.01**	**1.89**	**4.58**	**3.87**
Total Sugars (mg g^-1^_DW_) *	**129.36**	**121.18**	**164.45**	**142.04**	**140.08**	**104.42**

For each sugar, the mean values ± SE (n = 6) followed by different letters express significant differences between cultivars for the same CO_2_ treatment (a, b, c) or between CO_2_ treatments within the same cultivar (r, s). The ANOVAs for stachyose, glucose, fructose and galactose showed significant differences between cultivars for the same CO_2_ treatment, between CO_2_ treatments within the same cultivar, and for the interaction between genotype and growth CO_2_; that for arabinose showed significant differences between cultivars for the same CO_2_ treatment and between CO_2_ treatments within the same cultivar; those for sucrose and starch showed significant differences between cultivars for the same CO_2_ treatment and for the interaction; those for trehalose and mannitol showed significant differences between CO_2_ treatments within the same cultivar and for the interaction; and that for raffinose showed significant differences only between cultivars for the same CO_2_ treatment.

^*^ Values obtained using the mean values of each sugar compound.

Starch changes under high [CO_2_] were species-dependent, increasing by 69% in CL 153 while decreasing by 10 and 15% in Icatu and IPR 108, respectively.

Total non-structural carbohydrates (NSC) did not increase in any of the genotypes. In fact, NSC tended to decrease, particularly in IPR 108 (25%) and Icatu (13%).

### Photosynthetic pigments

**Figure 6:**
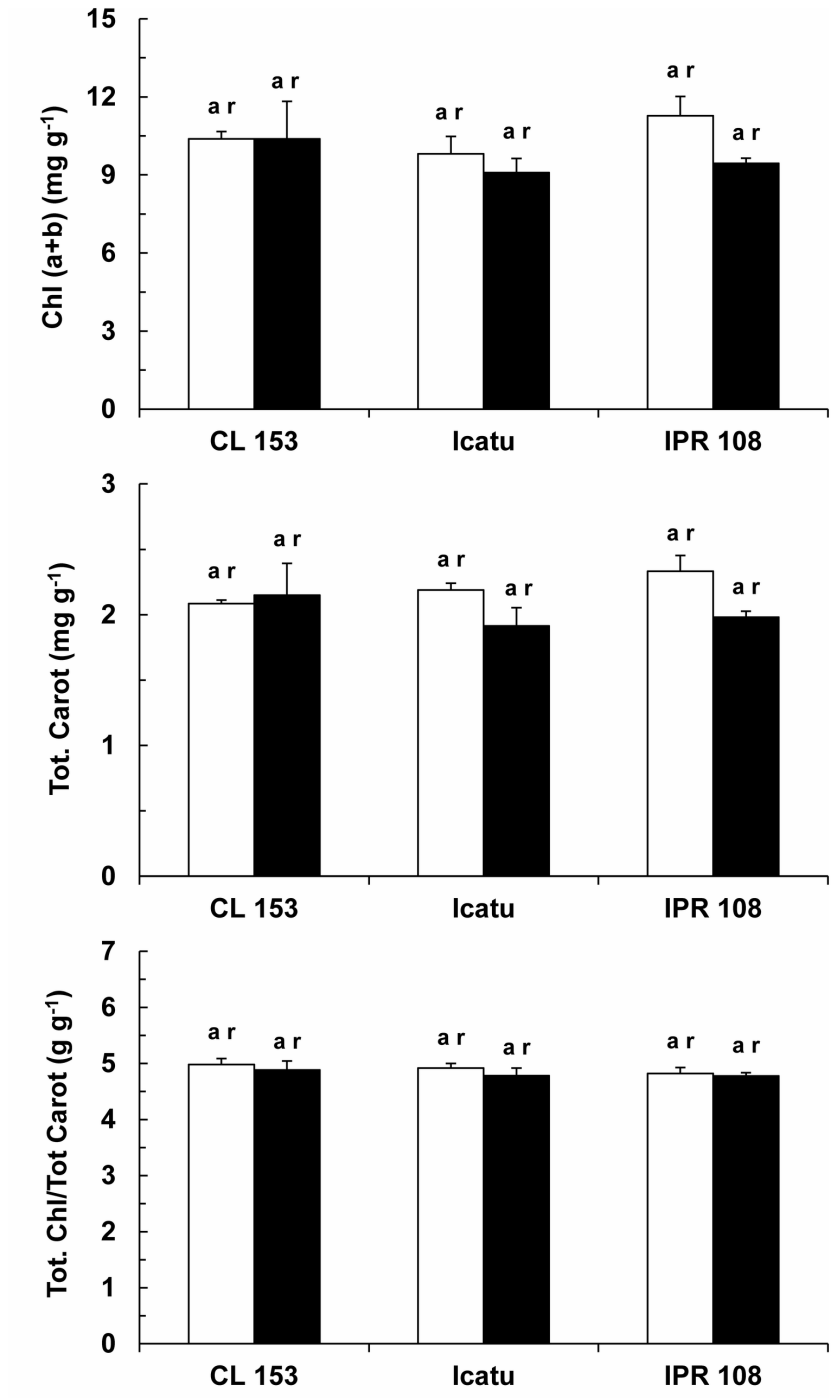
Photosynthetic pigment stability. Changes in the content of total chlorophylls (Chl *a*+*b*) and total carotenoids (Tot. Carot.), as well as their ratio (Tot Chl/Tot Carot), in the leaves of *C. arabica* (Icatu and IPR 108) and *C. canephora* (Conilon CL 153) grown under 380 (white bar) and 700 (black bar) μL CO_2_ L^-1^. For each parameter, the mean values + SE (n = 6-8) followed by different letters express significant differences between cultivars for the same CO_2_ treatment (a, b) or between CO_2_ treatments within the same cultivar (r, s).

Total chlorophylls, total carotenoids and their ratios did not differ significantly with respect to genotype or growth CO_2_ treatment (Figure 6).

### Cellular membrane permeability and quantification of chloroplast membrane lipids

**Figure 7:**
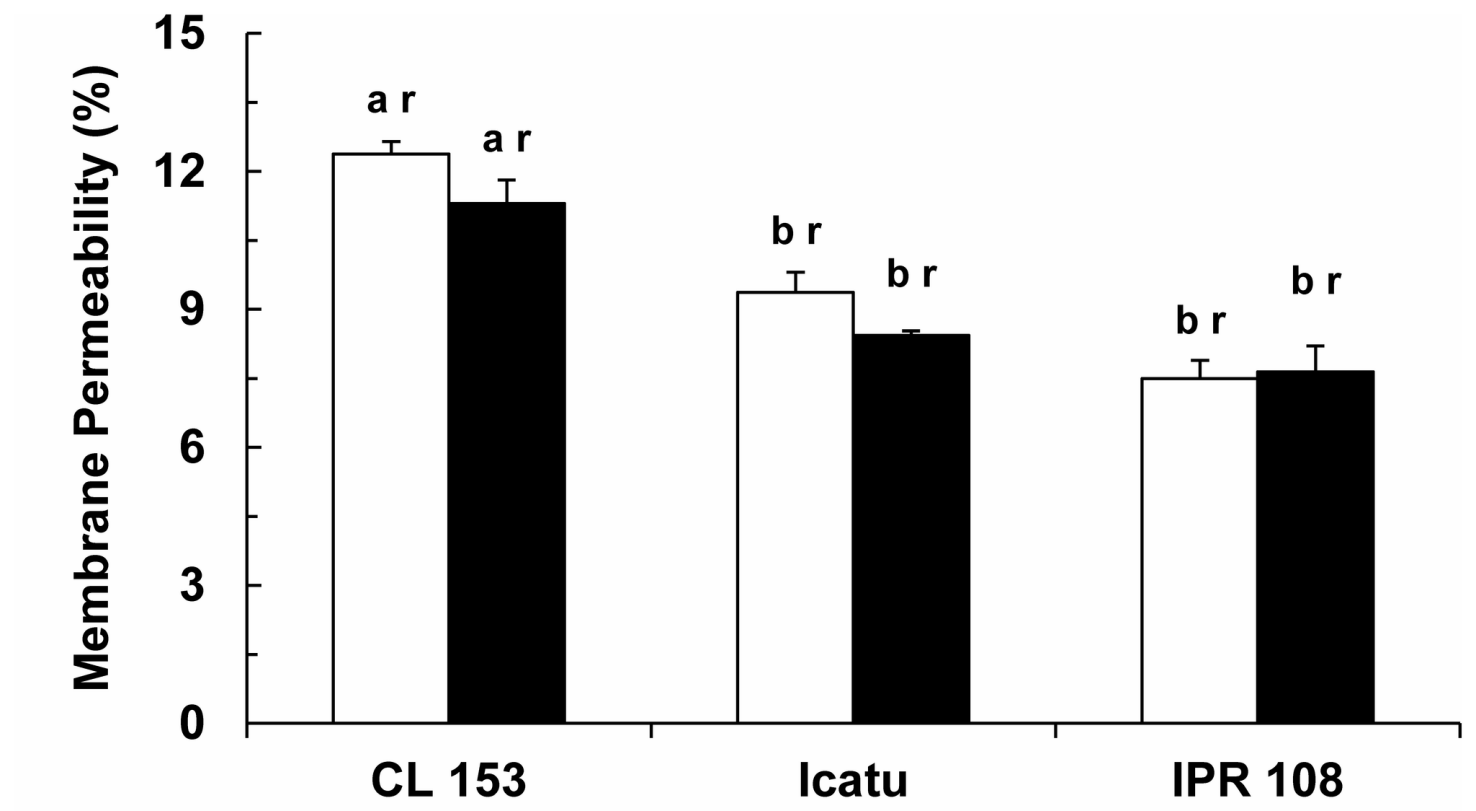
Cellular membranes selectivity stability. Evaluation of membrane permeability in the leaves of *C. arabica* (Icatu and IPR 108) and *C. canephora* (Conilon CL 153) grown under 380 (white bar) and 700 (black bar) μL CO_2_ L^-1^. The mean values + SE (n = 5) followed by different letters express significant differences between cultivars for the same CO_2_ treatment (a, b) or between CO_2_ treatments within the same cultivar (r, s). The ANOVA for leakage showed significant differences between cultivars for the same CO_2_ treatment, between CO_2_ treatments within the same cultivar and for the interaction.

**Figure 8:**
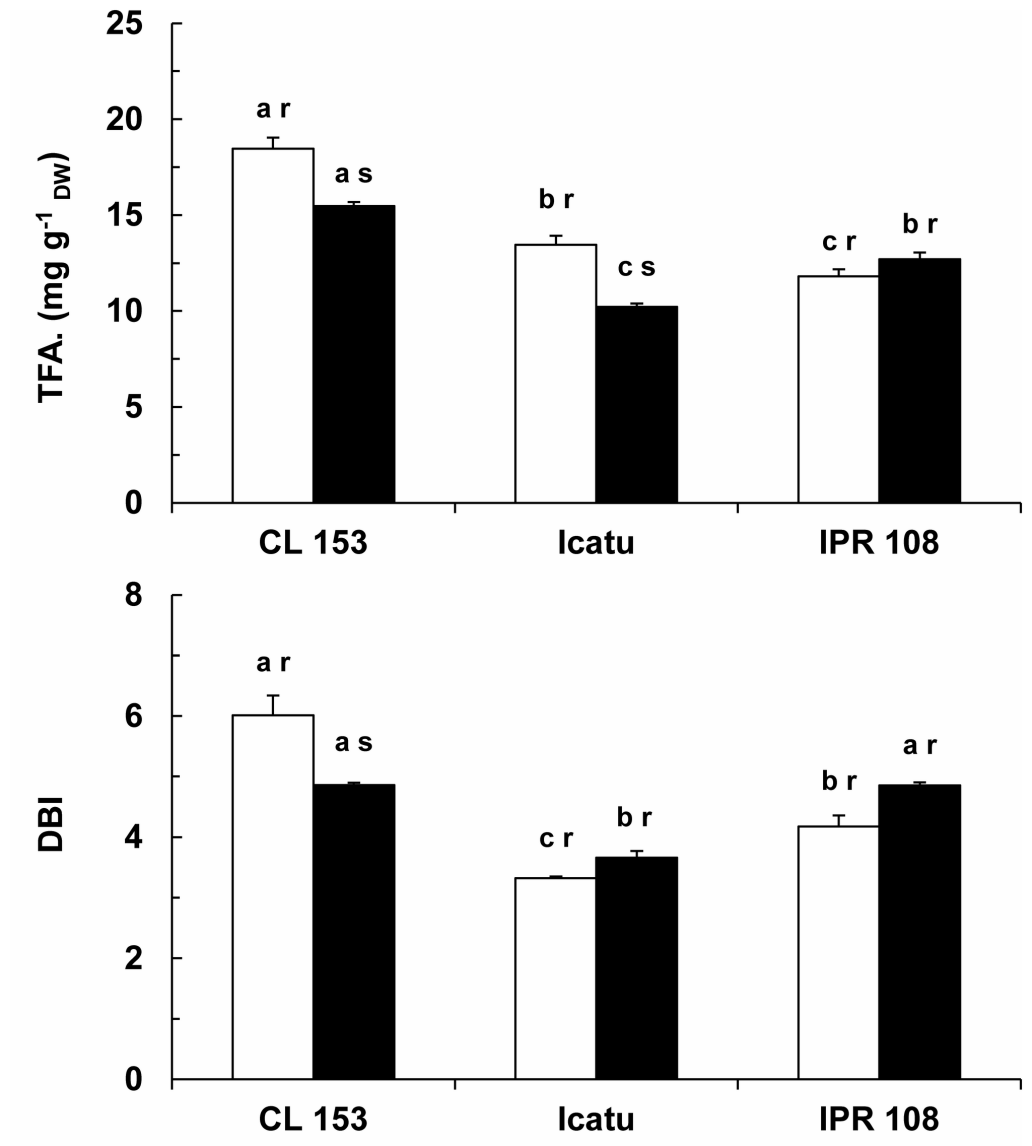
Dynamics of the lipid matrix from chloroplast membranes. Changes in the total fatty acid (TFA) content and unsaturation level (DBI) of the chloroplast membranes of leaves of *C. arabica* (Icatu and IPR 108) and *C. canephora* (Conilon CL 153) grown under 380 (white bar) and 700 (black bar) μL CO_2_ L^-1^. For each fatty acid, the mean values + SE (n = 4) followed by different letters express significant differences between cultivars for the same CO_2_ treatment (a, b) or between CO_2_ treatments within the same cultivar (r, s). The ANOVAs for TFA and DBI showed significant differences between cultivars for the same CO_2_ treatment, between CO_2_ treatments within the same cultivar, and for the interaction between genotype and growth CO_2_.

Among genotypes, some differences were noted in the cellular membrane leakage values at normal growth CO_2_, but these were not affected by enhanced growth [CO_2_] (Figure 7). In contrast, the lipid fraction displayed significant reductions of total fatty acid (TFA) content in CL 153 and Icatu (Figure 8). This reduction was accompanied by a decrease in the unsaturation level (lower values of the double bond index, DBI) in CL 153, although the opposite tendency was found for both Icatu and IPR 108. These changes in the DBI of CL 153 resulted from modifications in the weight of individual FAs (Table 5), as the contents of the two most important FAs, palmitic acid (C16:0) and linolenic acid (C18:3), followed opposite trends, with the latter decreasing significantly under high [CO_2_]. In the *C. arabica* genotypes, the weight of the individual FAs remained mostly unaltered, although minor changes occurred in C16:0 and C18:3, resulting in small increases in DBI. Additionally, the 3-trans-hexadecenoic acid (C16:1 *c*+*t*) tended to increase, albeit only significantly in IPR 108.

**Table 5:** Evaluation of the proportions of palmitic acid (C16:0), 3-trans-hexadecenoic acid (C16:1t), stearic acid (C18:0), oleic acid (C18:1), linoleic acid (C18:2) and linolenic acid (C18:3), from the chloroplast membranes of the leaves of *C. arabica* (Icatu and IPR 108) and *C. canephora* (Conilon CL 153) grown under 380 and 700 μL CO_2_ L^-1^.

**Genotype**	**CL 153**		**Icatu**		**IPR 108**	
**CO_2_ Treatment**	**380 μL L^-1^**	**700 μL L^-1^**	**380 μL L^-1^**	**700 μL L^-1^**	**380 μL L^-1^**	**700 μL L^-1^**
**<C16:0 (%)**	**2.54 ^c s^**	**5.62 ^b r^**	**4.57 ^b s^**	**7.56 ^a r^**	**5.33 ^a r^**	**4.80 ^c r^**
	**0.32**	**0.10**	**0.32**	**0.08**	**0.31**	**0.18**
**C16:0 (%)**	**21.9 ^c r^**	**24.6 ^b r^**	**32.3 ^a r^**	**27.8 ^a s^**	**26.8 ^b r^**	**24.8 ^b r^**
	**1.2**	**0.2**	**1.1**	**1.1**	**1.3**	**0.6**
**C16:1c+t (%)**	**3.26 ^a r^**	**2.10 ^b r^**	**2.31 ^ab r^**	**4.07 ^a r^**	**1.69 ^b s^**	**4.43 ^a r^**
	**0.55**	**0.81**	**0.28**	**0.34**	**0.38**	**0.51**
**C18:0 (%)**	**7.42 ^a r^**	**5.86 ^a r^**	**7.88 ^a r^**	**7.18 ^a r^**	**7.53 ^a r^**	**6.80 ^a r^**
	**0.27**	**0.19**	**0.99**	**0.52**	**0.11**	**0.29**
**C18:1c+t (%)**	**1.71 ^b s^**	**2.13 ^a r^**	**2.30 ^a r^**	**1.95 ^a r^**	**2.26 ^a r^**	**1.59 ^b s^**
	**0.01**	**0.02**	**0.18**	**0.16**	**0.03**	**0.03**
**C18:2 (%)**	**11.1 ^b s^**	**14.1 ^a r^**	**14.2 ^a r^**	**12.8 ^b r^**	**13.7 ^a r^**	**13.5 ^ab r^**
	**0.2**	**0.3**	**0.8**	**0.8**	**0.4**	**0.4**
**C18:3 (%)**	**52.1 ^a r^**	**45.6 ^a s^**	**36.4 ^c r^**	**38.6 ^b r^**	**42.7 ^b r^**	**44.1 ^a r^**
	**1.2**	**0.9**	**2.0**	**0.1**	**0.9**	**0.3**

For each fatty acid, the mean values ± SE (n = 4) followed by different letters express significant differences between cultivars for the same CO_2_ treatment (a, b, c) or between CO_2_ treatments within the same cultivar (r, s). The ANOVAs for all fatty acids showed significant differences between cultivars for the same CO_2_ treatment, between CO_2_ treatments within the same cultivar and for the interaction between genotype and growth CO_2_, except that for C18:0 that did not present a significant interaction effect.

## Discussion

### Long-term [CO_2_] enrichment did not provoke down-regulation of either stomatal conductance or photosynthesis

Stomatal size and density are key determinants of maximum *g*_*s*_ [38,54]. Here, we showed that the dichotomous behavior of these traits likely contributed to the low response of *g*_s_ to [CO_2_] enrichment in the tested genotypes (Table 1). These results agree with those of few studies that have reported invariant responses of *g*_s_ to elevated [CO_2_] in woody species [8,55,56]. Indeed, *g*_s_ has been systematically, but not universally, demonstrated to decrease when plants are grown under elevated [CO_2_] [8,16,17,22], although such decreases have been shown to occur to a lesser extent in shrubs and trees than in herbaceous annuals [8].

In the current study, no apparent photosynthetic down-regulation to long-term elevated [CO_2_] was observed. Rather, some components of the photosynthetic machinery were even up-regulated under high [CO_2_]. The present work presents compelling evidence that supports this conclusion. First, significant increases in P_n_ were found in either genotype when measured under elevated [CO_2_] (Figure 1). These increases were likely related not only to a higher carboxylation rate linked to the increase of CO_2_ as a substrate but also to the competitive inhibition of the oxygenation reaction of RuBisCo and the subsequent reduction of CO_2_ loss and energy costs associated with the photorespiratory pathway [7,8]. Second, despite the increase in P_n_, at least a partial photosynthetic and stomatal down-regulation could have occurred due to prolonged growth under high [CO_2_]. In fact, this was not the case; the P_n_ increase was independent of growth [CO_2_], as no significant differences existed between plants grown under normal or high [CO_2_] when measurements were performed at 380 or 700 µL CO_2_ L^-1^ (data not shown). This result indicates that the P_n_ stimulation was similar to the potential maximum stimulation obtained by the modeled P_n_/C_i_ response under current [CO_2_]. Third, A_max_ (assessed under saturating CO_2_ in the absence of diffusion-mediated limitations of photosynthesis) was unresponsive to the [CO_2_] treatments (Figure 2). Fourth, the acclimation of the key photosynthetic parameters (V_cmax_ and J_max_) was not observed (Table 2), in sharp contrast to previous studies [for review, see 4,14]. In fact, photosynthesis is known to shift from limitation by Rubisco carboxylation capacity to consume RuBP at low Ci (because CO_2_ is the substrate), to RuBP regeneration-limited rates at higher [CO_2_], related to the ability of thylakoid-dependent reactions to supply ATP and NAPDH [8,10], as well as to the capacity of starch and sucrose synthesis to utilize triose phosphates and subsequently regenerate P_i_ for photophosphorylation [21]. Irrespective of these assumptions, a modest average decrease (6%) of V_cmax_ has been observed in trees under high [CO_2_] [8], whereas a downward adjustment of J_max_ has been found less frequently [7]. The absence of the acclimation of J_max_ and V_cmax_ [9], or a positive acclimation to elevated [CO_2_], could also occur, shifting the investment of resources from RuBisCo to the processes supporting RuBP and Pi regeneration. Fifth, TPU, which may become limited under high [CO_2_] even without photosynthetic acclimation in trees [9], remained unchanged. This result suggests that P_n_ is not limited by the plant’s ability to synthesize (and use) starch and sucrose, using triose phosphates and regenerate P_i_ [21]. Sixth, the thylakoid electron transport involving both PSII and PSI, as well as the photosynthetic enzyme activities, were, overall, up-regulated under elevated [CO_2_] (Figure 3). Finally, no apparent up-regulation of the NSC pools was observed (see below). Considered together, these results suggest that if root growth is unrestricted by pot size [26,57], as it was in the present case, the coffee plant can sustain relatively high photosynthetic rates when growing under elevated [CO_2_] whenever water, temperature and nutrients are not limiting.

Another widely reported effect promoted by high growth [CO_2_] is the increase of WUE, often resulting from the maintenance of or decreases in g_s_ (and T_r_) in parallel to increases in P_n_, even when partial photosynthetic down-regulation occurs [8,14,17,19]. In this study, the increase in iWUE in CL 153 was mostly promoted by the enhancement of P_n_, as has been observed in grapevine [56] and in forest trees [9], whereas in Icatu and IPR 108 a tendency to lower g_s_ values also contributed to iWUE increase (Figure 1). Since changes in precipitation patterns and an increased frequency of drought episodes are predicted due to global warming [1,7], the improved leaf-level iWUE of plants grown under enhanced [CO_2_] could be of great interest, as it may help protect plants from water shortage. However, it must be noted that WUE increases due to transpiration (and g_s_) reductions at the leaf level do not necessarily lead to corresponding reductions in conductance and evapotranspiration at the canopy level [58]. Moreover, in the CL 153 plants, iWUE increased mostly due to a P_n_ upsurge under high CO_2_ conditions. Knowing that the predicted rise in temperature (and lower rainfall) that will accompany the increase in atmospheric CO_2_, is expected to increase the leaf-to-air vapor pressure (that is a crucial determinant of leaf transpiration, together with g_s_), thus, no large reduction of water use would be expected in CL 153, contrary to what could happens in the *C. arabica* genotypes where g_s_ tend to lower values.

### Metabolic machinery was adjusted in response to high [CO_2_]

In all tested genotypes, the most prominent effect of [CO_2_] was the up-regulation of the *in vitro* activities of key enzymes of carbon metabolism, Ru5PK (a crucial enzyme in the RuBP regeneration pathway), RuBisCo (Figure 4), MDH and PK (Figure 5), suggesting a reinforcement of the potential biochemical capacities of both photosynthesis and respiration under the present experimental conditions. Such reinforcement might prevent the down-regulation of photosynthesis, which has been associated with reductions in N allocation to RuBisCo, RuBP regeneration and proteins associated with electron transport [9]. However, the increase of total RuBisCo activity did not match the behavior of V_cmax_ in CL 153, which might be explained by a decreased RuBisCo activation state. Accordingly, increased activities of MDH and PK were also not accompanied by increases in respiration rates (R_d_), which remained unchanged in response to high [CO_2_] (Figure 2). Indeed, depending on the species, declining or unchanging R_d_ have commonly been observed under high [CO_2_] [see 3,16,59].

Increases in P_n_ under high [CO_2_] obviously require more energy and reducing equivalents, which is consistent with the increases in the potential electron transport capacity (Figure 3). These parallel increases were likely associated with the maintenance of a close functional balance between carboxylation and electron transport events (J_max_/V_cmax_), which seemed to be conserved among coffee genotypes and irrespective of growth [CO_2_] (Table 2). Similar results have been reported for some acacia species [17,60] and also in other plant species, where the unchanging J_max_/V_cmax_ ratio has been interpreted as reflecting an absence of resource redistribution among photosynthetic components [61]. Additionally, the reinforcement of photosynthetic components under high [CO_2_] was also unrelated to changes in photochemical efficiency as analyzed by Chl *a* fluorescence. Of particular interest, F_v_/F_m_, F_v_´/F_m_´ and q_P_ (Table 3) were quite stable across experimental conditions, as has also been found in grapevine [56]. Notably, these results agree with the absence of noticeable changes in the pools of total chlorophyll (Chl) and carotenoids (Figure 6), as has also been reported for Chl in other woody species [7,9]. Altogether, these data indicate a pattern of enhanced investment in key components of both photosynthetic and respiratory pathways under high growth [CO_2_] in coffee plants.

### Absence of non-structural sugars accumulation

The accumulation of non-structural carbohydrates (NSC) in leaves is one of the most pronounced and common responses of C_3_ plants to elevated [CO_2_], even in field-grown plants where rooting volume is unrestricted [7]. In coffee genotypes, several soluble sugars presented significant reductions under high growth [CO_2_] conditions, particularly trehalose, stachyose, galactose and mannitol, whereas fructose and glucose either remained stable or decreased, resulting in a reduction of both total soluble sugars and NSC contents (Table 4). Therefore, the absence of sugar accumulation in leaves would have contributed to the avoidance of photosynthetic down-regulation (Figure 1, Table 2), and further supports the lack of negative impacts on the photosynthetic enzymes studied (Figure 4). Similar NSC findings have been reported in young sunflower leaves [24], as well as in poplar, which was able to export over 90% of its photosynthate during the day and had a large capacity for temporary storage in starch, thus, maintaining its potential for C acquisition [62].

In coffee, the absence of an NSC increase could be related to the higher production of new leaves and plagiotropic (lateral) branches (somewhat modifying the architecture of the coffee plant), as well as flower and fruit production occurring up to twice a year (unpublished observation). This higher continuous production of vegetative and reproductive structures would maintain a higher consumption of photosynthates, contributing to an increased sink strength that would ultimately explain the absence of photosynthetic acclimation. Enhanced sink strength linked to the maintenance of the photosynthetic stimulation under CO_2_ enrichment has been observed in other species such as *Vernonia herbacea* [6], loblolly pine [63], sour orange [4] and grapevine [56], with positive yield implications.

### Preservation of cell membrane permeability and changes in chloroplast lipids

Under normal [CO_2_], TFA content and the degree of unsaturation (DBI) were slightly higher in the *C. canephora* genotype (Figure 8), as has also been previously reported [28]. Cell membrane permeability was not significantly modified due to growth [CO_2_] (Figure 7), but the lipid matrix of the chloroplast membranes was altered in terms of TFA content in CL 153 and Icatu. Additionally, variations in the major C16:0 and C18:3 FAs, which increased and decreased, respectively (Table 5), led to a decline in the lipid membrane unsaturation in CL 153, whereas the opposite tendency was found in the *C. arabica* genotypes, with possible implications for membrane fluidity [28,64]. Furthermore, C16:1*c*+*t* values tended to increase in the *C. arabica* genotypes, becoming somewhat higher than in CL 153. The C16:1t is a major and specific FA of the phosphatidylglycerol (PG) in chloroplasts [28,65,66]. Both PG and C16:1t contribute to preserve the thylakoid membrane’s organization of proteins and pigments and the optimal conformation of the D1 protein, stabilizing the photosystem complexes and allowing for efficient non-cyclic electron flow [66,67]. However, although some differences among genotypes were observed under high [CO_2_] concerning the lipid unsaturation degree and the composition in some FAs in the chloroplast membranes, no distinct impacts on plant function, e.g., changes in electron transport, could be detected.

### No clear differences between *C. arabica* and *C. Canephora* genotypes were observed in response to elevated [CO_2_]

No clear species-dependent responses to elevated [CO_2_] were found, as the three genotypes displayed similar trends for most of the collected data. Nonetheless, some differences seemed to exist between the genotypes. For example, IPR 108 was somewhat less responsive for most parameters, Icatu presented the highest increases on V_cmax_ and J_max_, whereas CL 153 showed the more stable V_cmax_ and J_max_ values (Table 2), the highest thylakoid electron transport capability (Figure 3) and starch increase (Table 4), accompanied by a reduction in the unsaturation of chloroplast membrane lipids (Figure 8, Table 5). Regardless of these differences and genotypes, our data suggest that coffee can successfully cope with high [CO_2_] under optimal conditions (water, temperature and nutrient availability), but further studies are necessary to evaluate its responses to a broader scenario of climatic changes, including altered water availability and high temperatures.

## Conclusions

Under enhanced growth [CO_2_], no down regulation of g_s_ and P_n_ was observed in the current study under unrestricted conditions of water, nutrients and root development. Stomatal density and size showed dichotomous behaviors, decreasing and increasing, respectively, whereas g_s_ exhibit no significant response to elevated [CO_2_]. Most importantly, changes in P_n_ largely governed the significant rise in the iWUE under elevated [CO_2_], although in the *C. arabica* cultivars g_s_ may have somewhat contributed. No negative impacts on the P_n_, J_max_ and V_cmax_ values were observed, a result most likely linked with the absence of NSC accumulation. Considering the stable environmental conditions of this experiment, this absence of NSC accumulation may be related to a sufficiently large sink capacity of the plants due to their continuous production of vegetative (branches and leaves) and reproductive (flowers and fruits) structures, as observed during the one-year experiment. Furthermore, adjustments in the metabolic machinery of the photosynthetic (and respiratory) pathways included increases in PSs, RuBisCo, Ru5PK, MDH and PK activities, contributing to the somewhat higher values of V_cmax_ (Icatu, IPR 108) and J_max_ (Icatu). These adjustments did not, however, impact the efficiency of PSII functioning (F_v_/F_m_, F_v_´/F_m_´), energy driven to photochemical events (q_P_), photosynthetic pigments or membrane selectivity, under these moderate irradiance levels. Some changes in the degree of unsaturation of the chloroplast membranes were found (a decrease in CL153 and a tendency to increase in the *C. arabica* genotypes) due to changes in the most prevalent fatty acids, palmitic acid (C16:0) and linolenic acid (C18:3). Yet, these changes were not clearly related to photosynthetic functioning. Finally, despite the differences found in some parameters, no clear species-dependent response was found in relation to growth [CO_2_].
